# Supplementary figures and images for: Insect detect: An open-source DIY camera trap for automated insect monitoring
Source: PLoS One. 2024 Apr 3;19(4):e0295474. doi: 10.1371/journal.pone.0295474 (PMC10990185; doi:10.1371/journal.pone.0295474)

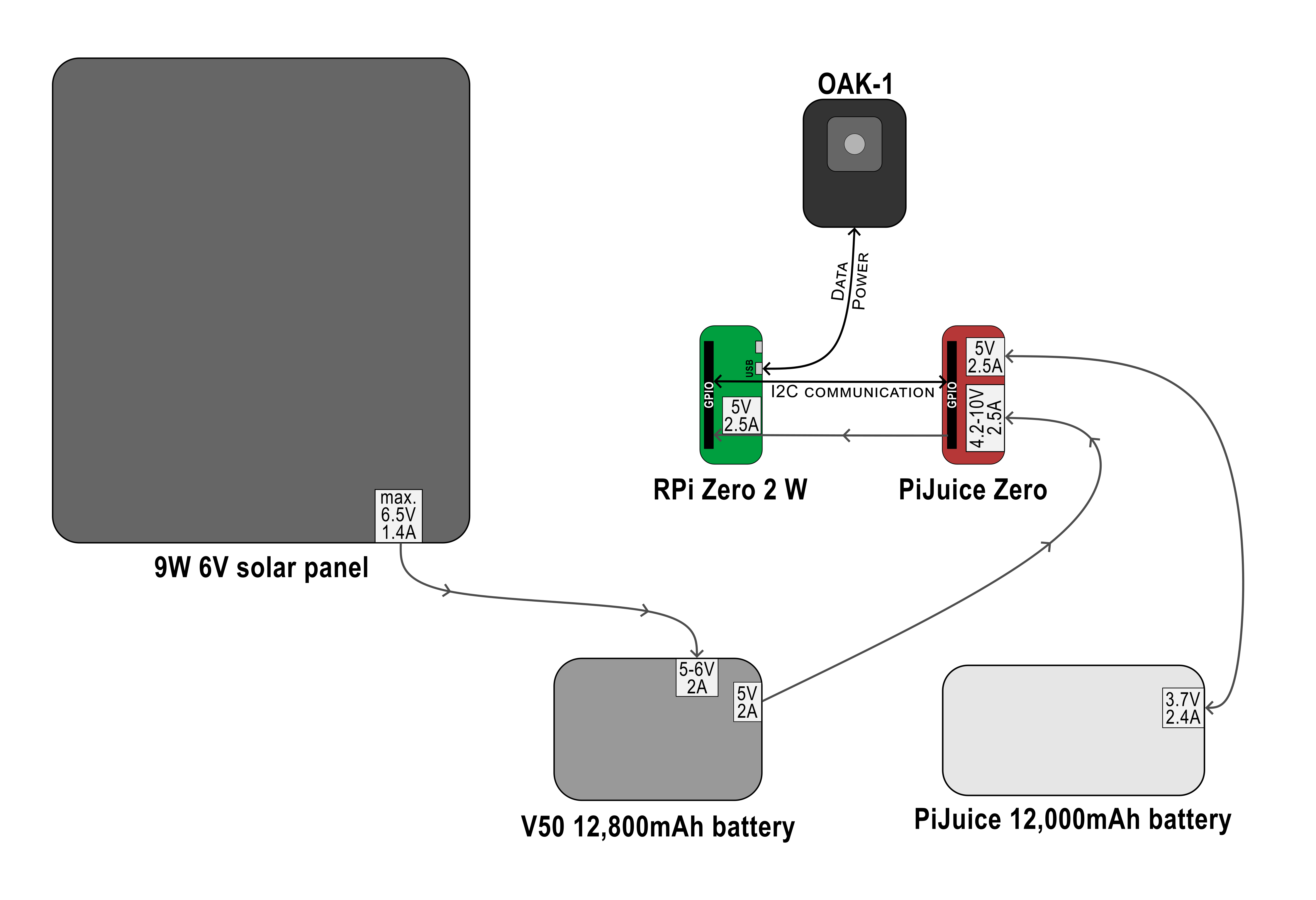

Supplement: S1 Fig — Nominal voltage is shown for the PiJuice 12,000 mAh battery. (TIFF) [file pone.0295474.s004.tiff]

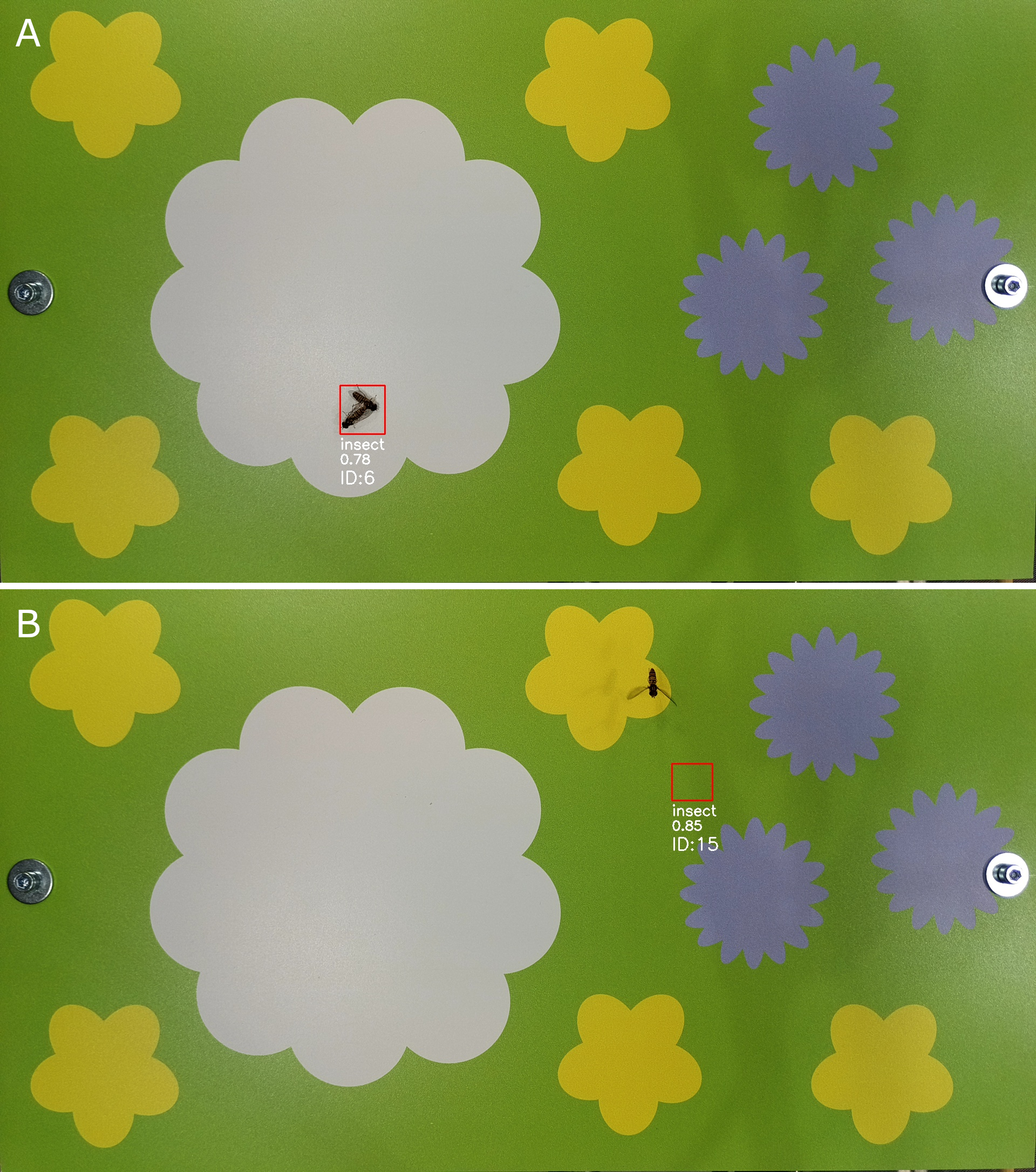

Supplement: S2 Fig — (A) The same tracking ID is assigned to insects coming close to each other. (B) A fast-moving insect is not correctly tracked, with the risk of a new tracking ID being assigned to the same individual. (TIFF) [file pone.0295474.s005.tiff]

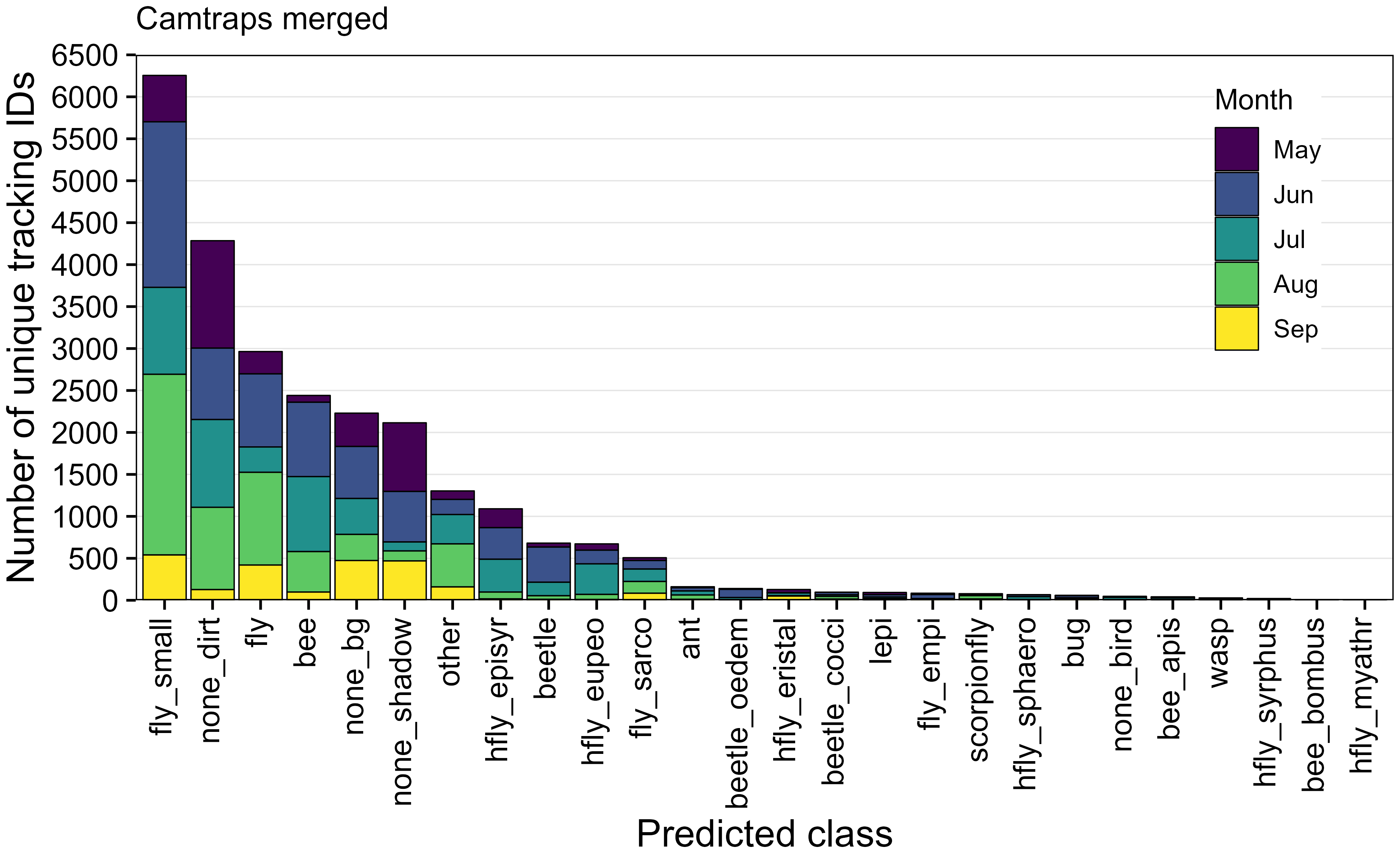

Supplement: S3 Fig — Merged data from all five camera traps deployed from mid-May to mid-September 2023. All tracking IDs with less than three or more than 1,800 images were removed. (TIFF) [file pone.0295474.s006.tiff]

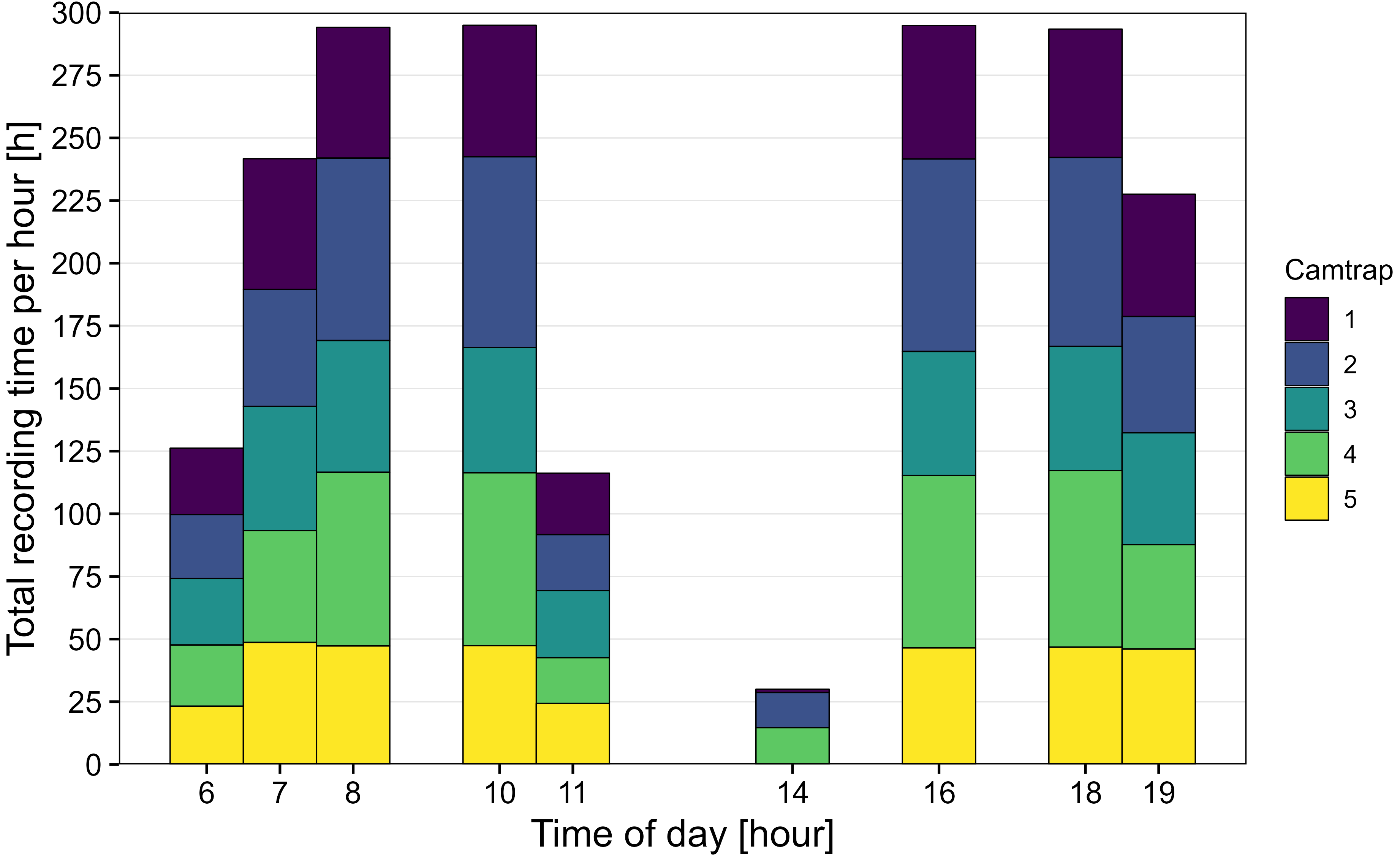

Supplement: S4 Fig — Merged data from all five camera traps deployed from mid-May to mid-September 2023. (TIFF) [file pone.0295474.s007.tiff]

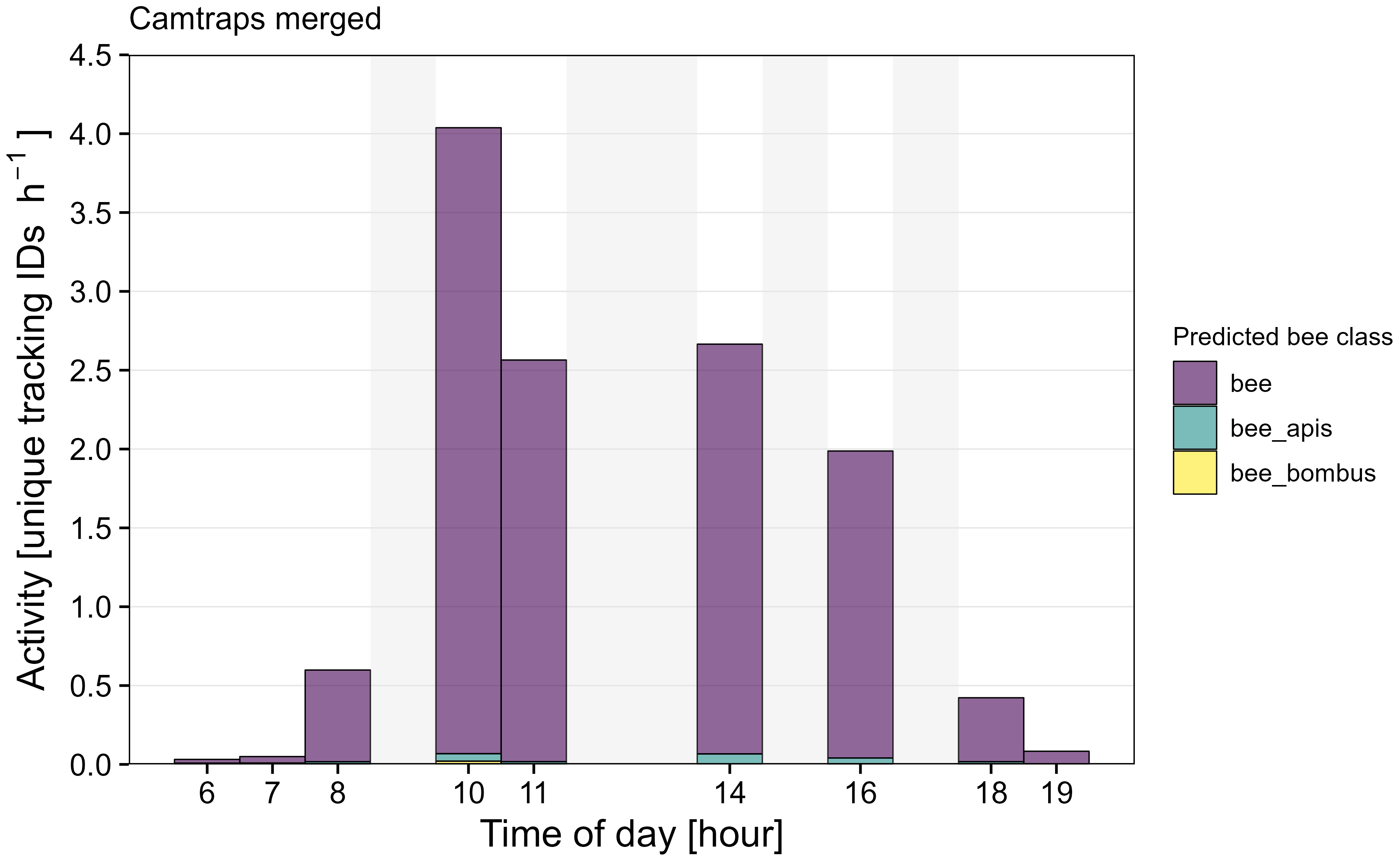

Supplement: S5 Fig — Merged data from all five camera traps deployed from mid-May to mid-September 2023. Shaded areas indicate hours without recordings. All tracking IDs with less than three or more than 1,800 images were removed. (TIFF) [file pone.0295474.s008.tiff]

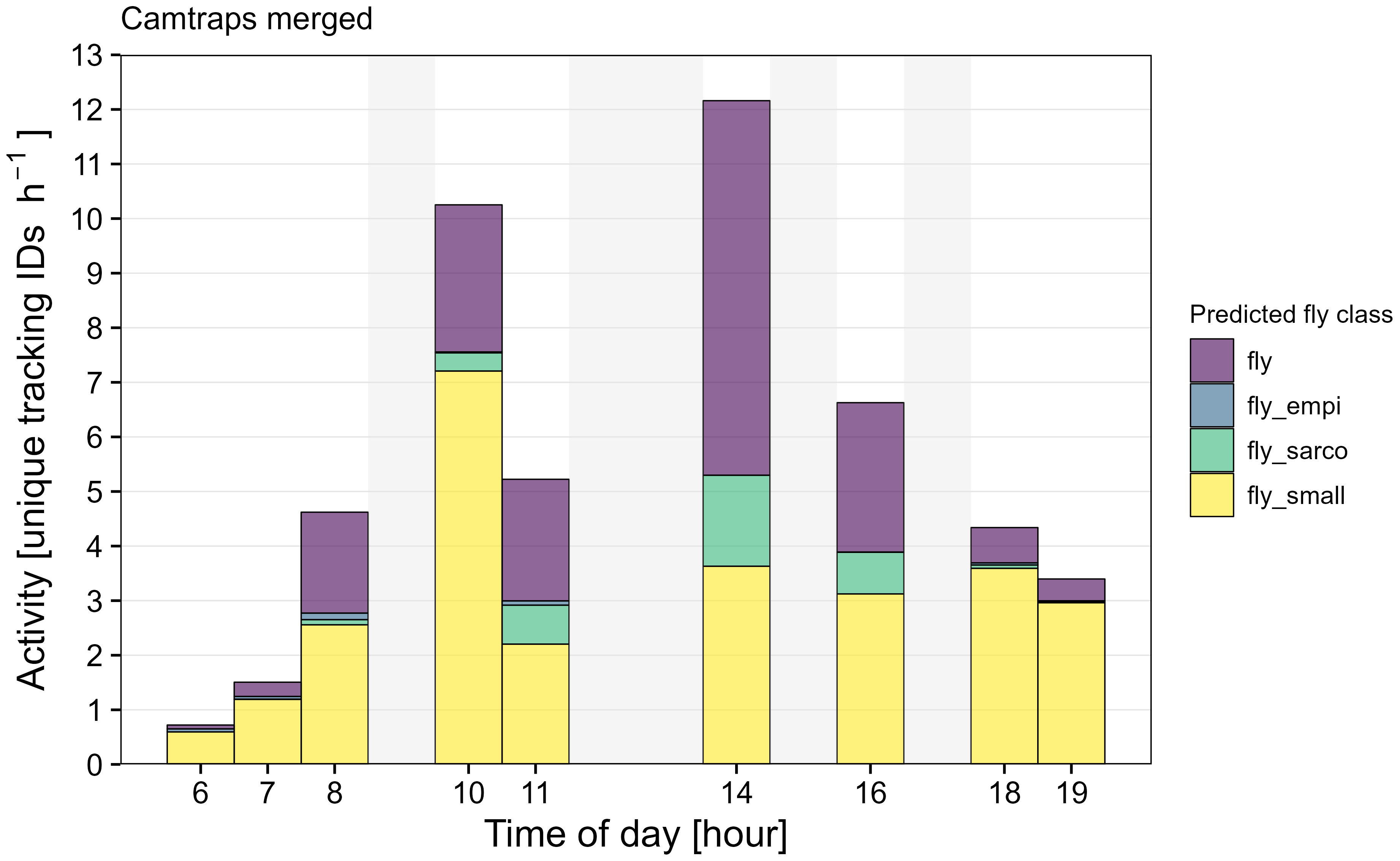

Supplement: S6 Fig — Merged data from all five camera traps deployed from mid-May to mid-September 2023. Shaded areas indicate hours without recordings. All tracking IDs with less than three or more than 1,800 images were removed. (TIFF) [file pone.0295474.s009.tiff]
