# Supplementary material for: Insect detect: An open-source DIY camera trap for automated insect monitoring
Source: PLoS One. 2024 Apr 3;19(4):e0295474. doi: 10.1371/journal.pone.0295474 (PMC10990185; doi:10.1371/journal.pone.0295474)
Supplement: Attachment — Submitted filename: Response_to_reviewers.docx [file pone.0295474.s011.docx]

PONE-D-23-38812R1

Insect Detect: An open-source DIY camera trap for automated insect monitoring

**Response to Reviewers**

Dear Prof. Mansour,

Thank you for giving us the opportunity to submit the second revision of our draft of the manuscript “Insect Detect: An open-source DIY camera trap for automated insect monitoring” for publication in PLOS ONE. We also want to thank Reviewer #1 for the detailed feedback and very valuable comments to improve our second revised draft of the manuscript. We want to thank Reviewer #2 for accepting our previous revisions and the encouraging words. We have incorporated the suggestions or included detailed arguments if a suggestion was not considered for the revised version of the manuscript. Please see below for a point-by-point response to the reviewers’ comments. All line numbers in the response refer to the revised manuscript file with tracked changes.

Sincerely,

Maximilian Sittinger, Johannes Uhler, Maximilian Pink, Annette Herz

**Authors** **changes to the manuscript:**

As the corresponding author (Maximilian Sittinger) has recently changed his position, we updated the E-mail address and added the current address in lines **12-14**.

We moved the general software description, previously in lines **168-172**, under the “Software” heading in lines **157-161**. We also added information about two new settings that were recently implemented in lines **161-165**.

To account for recent updates of the Python code that is running on the camera trap hardware, we changed “Script node” to “Sync node” in **Fig. 2**. We also changed the text accordingly in line **212**.

In line **237**, we deleted “[…] (OAK-1)”, which was a misleading additional information, as the cropping is done on the Raspberry Pi. “on device” now refers to the camera trap in general.

**Reviewer #1:**

Comments to the Authors:

The paper titled "Insect Detect: An Open-Source DIY Camera Trap for Automated Insect Monitoring" represents a commendable effort, offering a hardware solution for insect counting in natural environments and their classification into taxonomic orders, thereby providing significant value. This contribution to the scientific community, encompassing both the hardware solution and the open-source code, underscores its importance. However, regrettably, the text itself presents challenges in readability. It's not the language per se, but rather the overall structure and organization of section headings that can be perplexing. In my view, the authors should strongly consider revising some section headings and streamlining certain portions of the paper to enhance its accessibility and, consequently, its impact as a valuable scientific resource. Below, I outline specific questions regarding these aspects.

1. Figure 5 could benefit from improvement. It's unclear why there are x and y dimensions labeled for all images, especially considering they are of different sizes. Shouldn't the images be resized to a consistent size? Removing the axes would free up space, allowing for slightly larger images and thus improving visibility.

**Authors response:** Thank you for your suggestion. The individual images in Fig 5 show an example of the respective class from the dataset for classification model training. It is correct that all images are resized to the same size during classification model training and inference (in our model 128x128 pixel). However, all images shown in Fig 5 were automatically captured with the camera trap by cropping the detections (bounding box area) from synchronized HQ frames, which results in different image sizes, depending on the size of the bounding box (e.g. smaller for “ant”, bigger for “lepi”). To improve the clarity and comprehensibility of Fig 5, we decided to resize all example images to the same dimension. To still give the readers the important information about the original image sizes (as captured by the camera trap), we added x- and y-axis labels representing the original pixel values. We believe that the importance of this information outweighs the improved visibility of slightly larger images without the axes.

2. In the paragraph beginning at line 285, you mention that the dataset is divided into 80% for training, 10% for validation, and 10% for testing. Could you clarify how this division is achieved? Is it done randomly or as 80%, 10%, 10% for each class?

**Authors response:** Thank you for finding this important missing information. We added “[…] randomly […]” in line **296** to make it clear that the dataset was split randomly. This still means that all images of each class are split into the train (70%), validation (20%) and test (10%) subsets with the same ratio. However, as we are dealing with an imbalanced dataset, this results in different numbers of images per class in each subset (see also Table 2 for the test dataset). As this information was also missing for the dataset for detection model training, we added “[…] randomly […]” also in line **182**.

3. In the paragraph starting at line 285, you mention that you are utilizing the YOLOv5 classifier. Could you provide insights into any differences between training a typical ResNet50 versus a YOLOv5 ResNet50? Your commentary on this topic would be greatly appreciated.

**Authors response:** This is indeed an interesting point. In fact, we only use a slightly modified version of the Python script for classification model training from the YOLOv5 repository (<https://github.com/maxsitt/yolov5/blob/master/classify/train.py>) and not a specific “YOLOv5 classifier”. The ResNet50 and EfficientNet-B0 models are taken from the torchvision.models subpackage and were pre-trained on the ImageNet dataset (more info here: <https://github.com/ultralytics/yolov5/tree/master#classification> under “Classification Checkpoints”). In theory, the YOLOv5 script for classification model training supports all available PyTorch implementations of the (optionally pre-trained) classification model architectures from torchvision.models (see here: <https://pytorch.org/vision/stable/models.html#classification>). As a more thorough comparison of many different classification model architectures was out of scope for this manuscript, we only focused on some of the pre-trained weights published together with the YOLOv5 v6.2 release (<https://github.com/ultralytics/yolov5/releases/v6.2>). We are currently working on optimizing this classification model training and inference workflow by comparing more architectures. Results from these tests will be published in an upcoming paper. To come back to the reviewer’s question: the ResNet50 and EfficientNet-B0 are the official PyTorch implementations of both model architectures, available in the torchvision package and pre-trained on the ImageNet dataset. To avoid overcomplicating this topic, we are not including this additional information in the manuscript as it would probably only confuse most of the (non-professional) readers and would not add important information to our already described workflow.

4. In the paragraph starting at line 285, you mention using an image size of 128x128 for insect classification and 320x320 for initial insect detection. While the results are promising at these resolutions, could you comment on whether using a larger image size would further improve results? Additionally, considering hardware constraints, especially if this is not being done on the Pi-module, how does this impact your choice of image size?

**Authors response:** Thank you for this question. First, regarding the resolution of 320x320 pixel for insect detection (and tracking) running on the device in real time: We explain in lines **203-205** that this downscaled resolution (LQ frames) increases the inference speed of the detection model and thereby the accuracy of the object tracker. We tested different resolutions for our use case and 320x320 pixel resulted in the optimal balance between accuracy and speed. Depending on the specific use case (background, distance from camera to object, object size, object speed etc.) the optimal resolution could be lower or higher than what we are using. We mention multiple times in the Introduction and Discussion that users can train models on their custom datasets that are more adapted to their specific use case. By providing detailed documentation and Google Colab notebooks we enable also non-professional users to train their custom models, optionally on a different image resolution that is better suited for their use case (e.g. even lower resolution to increase the model’s inference speed). Second, regarding the resolution of 128x128 pixel for insect classification: we tested different image resolutions (see also S2 Table) for classification model training/inference. Resizing the insect images (= cropped detections) to a resolution of 128x128 pixel means that most of the images in our training dataset were upscaled. This led to better results compared to downscaling more of the images. In the Google Colab notebook for classification model training, we give users the option to calculate the metrics of their custom image dataset (<https://colab.research.google.com/github/maxsitt/insect-detect-ml/blob/main/notebooks/YOLOv5_classification_training.ipynb#scrollTo=aVZgGZtc8skH>). We recommend using the 90th percentile of the image sizes (divisible by 32) as reference point to set the image size for model training (and later inference). However, this is only an estimated reference point and it is still required to test different resolutions to find the optimal model when training on a new custom dataset. A different aspect is the HQ frame resolution that is used for cropping the detections. We explain in lines **217-225** that a higher resolution can increase classification accuracy but can also decrease tracking accuracy as the inference speed of the detection model is reduced.

5. I find the section headings confusing, particularly regarding the processing pipeline. Starting with section 2.2 on software, why is insect detection (2.2.1) not within the processing pipeline (2.2.2)? In my opinion, the software section should be renamed "Processing Pipeline," or better yet, remove the hardware and software distinctions entirely and replace them with more thematically fitting headings, such as 2.1. Pimodule - Insect Detect and 2.2. Processing Pipeline.

**Authors response:** Thank you for your suggestion. We built the entire workflow (from on-device detection to metadata post-processing) in a modular way, so that users can change their specific settings or deployed models at different stages of this workflow, without affecting previous/subsequent steps. For example, own custom models can be used for the insect detection without the need to change the on-device processing pipeline. Also, different classification models/workflows can be used for insect images captured with the camera trap. It is also possible to use the classification model/workflow on other images that were not necessarily captured with our proposed camera trap system. To indicate these different “workflow modules”, we used a subsection header for each step. We added “On-device […]” to the subsection headings in line **167** and **201** to clarify the distinction to the subsequent classification and metadata post-processing that is not happening on the camera trap.

6. In line 183, Table 1 there are results for insect detection in the methods section. Shouldn't these results be in the results section instead?

**Authors response:** Thank you for your suggestion. While the metrics of the detection models (and the classification model) are more often shown in the Results section in similar research articles, we focus more on non-professional readers and best possible clarity and accessibility. By already showing the model metrics in the respective subsections of the Materials and methods section, potential users are guided through the whole workflow in a more logical and cohesive way, without having to jump between sections. From our point of view, it is also easier to understand the subsequent processing steps if information about the model performance is given before explaining the on-device processing pipeline in more detail. In contrast to similar research works, we do not focus so strongly on the performance of the presented models and see them more as a baseline or example for interested user to train their own models on custom datasets. While we agree that this is probably a more unconventional way to present model metrics, we would like to keep them in the Materials and methods section for the mentioned reasons of increased accessibility.

**Reviewer #2:**

This paper introduces an automated, do-it-yourself camera trap system for studying flower-visiting insects. The system comprises two key components: a real-time camera equipped with a deep learning-based object detector. This detector identifies and captures images of insects landing on an artificial platform. Additionally, an insect classification model analyzes the captured images to identify the species of each insect.

The authors have adequately addressed the comments and concerns raised in the previous review, incorporating the necessary changes into the manuscript. I encourage the authors to continue updating and maintaining the documentation and software associated with this research, as it provides a valuable tool for researchers and citizen scientists engaged in insect monitoring studies.

**Authors response:** Thank you for your comment! We will continuously update and maintain the software and documentation to implement new features, further improve the detection and classification models and keep all requirements up to date.
